# Midwives and Infantile Ophthalmia

**Published:** 1902-02-01

**Authors:** 


### Midwives and Infantile Ophthalmia.

The Bill for the Registration of Midwives, or some measure equivalent to it, is to be reintroduced during the present session of Parliament; and as a good place has been obtained for it in the ballot, there is some probability that, in a more or less modified form, it may become law. Into the merits or demerits of its several provisions we do not at present propose to enter, and we only mention it in order to direct attention to the precautions taken in Germany, and in some of the United States, for the purpose of procuring the prompt treatment, by qualified medical practitioners, of every occurring case of infantile purulent ophthalmia.

In Germany, according to Dr. Minis Hays, special precautions are now taken against neglect of this disease, and to that end midwives are expressly prohibited by law from treating any affection of the eyes or eyelids, however slight. On the appearance of the first symptom they are required to represent to the parents or others in charge that medical assistance is urgently required, or, if necessary, they are themselves to report to the local authorities and the district doctor. Neglect of these regulations renders them liable to punishment. The States of New York, Rhode Island, Maine, Minnesota, Ohio, Maryland, Missouri, Massachusetts, Connecticut, Pennsylvania, New Jersey, Iowa, and South Carolina have within the last few years successively enacted laws declaring that, if one or both eyes of an infant become inflamed or swollen or reddened at any time within two weeks of its birth, it shall be the duty of the midwife or nurse having charge of such infant to report in writing, within six hours, to the health officer, or to some legally qualified physician of the city, town, or district in which the parents of the infant reside, the fact that such inflammation or swelling or redness of the eyes exists. The penalty for failure to comply with the law is a fine not to exceed 200 dollars, or imprisonment not to exceed six months, or both.

The justification for legislation of this character, which might very easily be included in any Act providing for the registration of midwives, is that the purulent ophthalmia of infancy is perfectly harmless when properly treated at an early period, that it destroys sight in about 50 per cent. of the cases in which it is neglected or treated improperly, and that it is sufficiently prevalent to be the main source of supply to all the educational institutions for the blind, or, in other words, to be the chief, and, except for inherited syphilis, almost the sole cause of blindness in early life. According to Magnus, Fuchs, and other authorities on the subject, more than one quarter of the entire blindness of the civilised world is caused by infantile ophthalmia; and such blindness, beginning with life itself, relegates the patient, in the great majority of instances, from the position of a producer to that of a comparatively useless consumer of the production of others. Taking only the pecuniary aspect of the question, Dr. Hays points out that, of the 50,000 blind persons in the United States, it must be assumed that 15,000 lost their sight from neglected purulent ophthalmia in infancy, and then, taking the cost of maintenance of a single person, as shown by the balance-sheets of the best regulated American asylums, to be at least $132 a year, and the average earnings of a single able-bodied person to be $1 a day, he shows the total loss to the commonwealth of the United States, from the ravages of this disease, to amount to $7,500,000 annually, or, approximately, a million and a half sterling. That amount is something like the yield of a penny in the pound income-tax in Great Britain. According to the census of 1891, the blind in England and Wales amounted to 809 per million of population, and, assuming this proportion to have remained unaltered, the total number of them would now, in round numbers, be 26,000, of whom 7,500 would be the victims of the disease we are considering. We have not at hand any materials from which to estimate the cost of maintenance of these unfortunates, as contrasted with the sums which, if they had retained vision, they might have been expected to earn; but we have probably said enough to show what are the demands of humanity in relation to the subject. It is one with which the British Government has hitherto refused to interfere, for alleged reasons little creditable to those by whom they have been advanced; but it may fairly be contended that the passage of any Act for the registration of midwives, and for the regulation of their practice, would afford an admirable opportunity for dealing with a great and admitted evil.

